# Ixr1 Regulates Ribosomal Gene Transcription and Yeast Response to Cisplatin

**DOI:** 10.1038/s41598-018-21439-1

**Published:** 2018-02-15

**Authors:** Ángel Vizoso-Vázquez, Mónica Lamas-Maceiras, M. Isabel González-Siso, M. Esperanza Cerdán

**Affiliations:** Universidade da Coruña, Grupo EXPRELA, Centro de Investigacións Científicas Avanzadas (CICA), Facultade de Ciencias, 15071 A Coruña, Spain

## Abstract

Ixr1 is a *Saccharomyces cerevisiae* HMGB protein that regulates the hypoxic regulon and also controls the expression of other genes involved in the oxidative stress response or re-adaptation of catabolic and anabolic fluxes when oxygen is limiting. Ixr1 also binds with high affinity to cisplatin-DNA adducts and modulates DNA repair. The influence of Ixr1 on transcription in the absence or presence of cisplatin has been analyzed in this work. Ixr1 regulates other transcriptional factors that respond to nutrient availability or extracellular and intracellular stress stimuli, some controlled by the TOR pathway and PKA signaling. Ixr1 controls transcription of ribosomal RNAs and genes encoding ribosomal proteins or involved in ribosome assembly. qPCR, ChIP, and 18S and 25S rRNAs measurement have confirmed this function. Ixr1 binds directly to several promoters of genes related to rRNA transcription and ribosome biogenesis. Cisplatin treatment mimics the effect of *IXR1* deletion on rRNA and ribosomal gene transcription, and prevents Ixr1 binding to specific promoters related to these processes.

## Introduction

The high-mobility group (HMG) proteins, present in almost all metazoans and plants, were discovered as nuclear factors over 40 years ago^1^. The HMG-box that characterizes the HMGB subfamily^2^ comprises 3 α-helices folded into an L-shaped configuration, in which the concave surface binds to the minor groove of DNA^3^. They act in the nucleus as non-histone architectural-chromatin proteins, having regulatory functions in replication, transcription and DNA repair^4^. HMGB proteins also bind with high affinity to distorted DNA structures, such as 4-way junctions, bulges, kinks, and modified DNA containing cisplatin adducts^5^.

Ixr1 (Intrastrand cross (X)-link recognition, formerly called Ord1, for Oxygen/oxidase regulation defective) is a *Saccharomyces cerevisiae* HMGB protein that regulates the transcription of genes involved in the response to normoxia-hypoxia changes^6–10^. Ixr1 also binds to cisplatin-DNA adducts with high affinity^11^. A model to explain the dual role of Ixr1 in transcriptional regulation and recognition of cisplatin-DNA adducts has recently been proposed^12^.

Cisplatin is used in cancer chemotherapy^13^, however cancerous cells usually acquire resistance against the drug soon after the initial treatment, thus limiting its effectiveness^14^. The molecular mechanisms of cisplatin cytotoxicity and acquired resistance in mammals have been thoroughly reviewed^15^. Yeast has been used as an easy-to-handle eukaryotic model to find genes related to cisplatin responsiveness and *IXR1* deletion leads to a hyperresistance^16–18^. *IXR1* mutation also favors the rate of spontaneous mutagenesis mediated by replication errors^19^. The hypothesis that Ixr1 and other HMG-domain proteins might block repair of the major cisplatin-DNA adducts *in vivo*, thus inducing cell death, was postulated over 20 years ago^20^. It is supported by the evidence that *IXR1* deletion does not increase resistance of *S*. *cerevisiae* cells to cisplatin that already have mutations in the *RAD* genes related to DNA-repair^20^. A more recent model suggests that elimination of Ixr1 pre-activates the genome integrity checkpoint above basal levels, thereby increasing DNA repair and cisplatin resistance^21^. Ixr1 is also required for the maintenance of an adequate supply and balance of dNTPs for DNA synthesis and repair^22^.

We have analyzed transcriptomes to compare the regulatory roles of Ixr1 in absence or presence of cisplatin. Ixr1 regulates in yeast other transcriptional factors, which respond to external stimuli and control cell growth and proliferation. A number of approaches - qPCR, ChIP to CHIP, and quantification of 18S and 25S rRNAs - confirm the function of Ixr1 in the control of ribosome biogenesis. Connections between this control, the TOR signaling pathway and the effects of cisplatin are discussed.

## Results

### Ixr1 controls genes related to hypoxic response, oxidative stress, metabolism of sulfur containing compounds, DNA damage response and ribosome biogenesis

**Figure 1:**
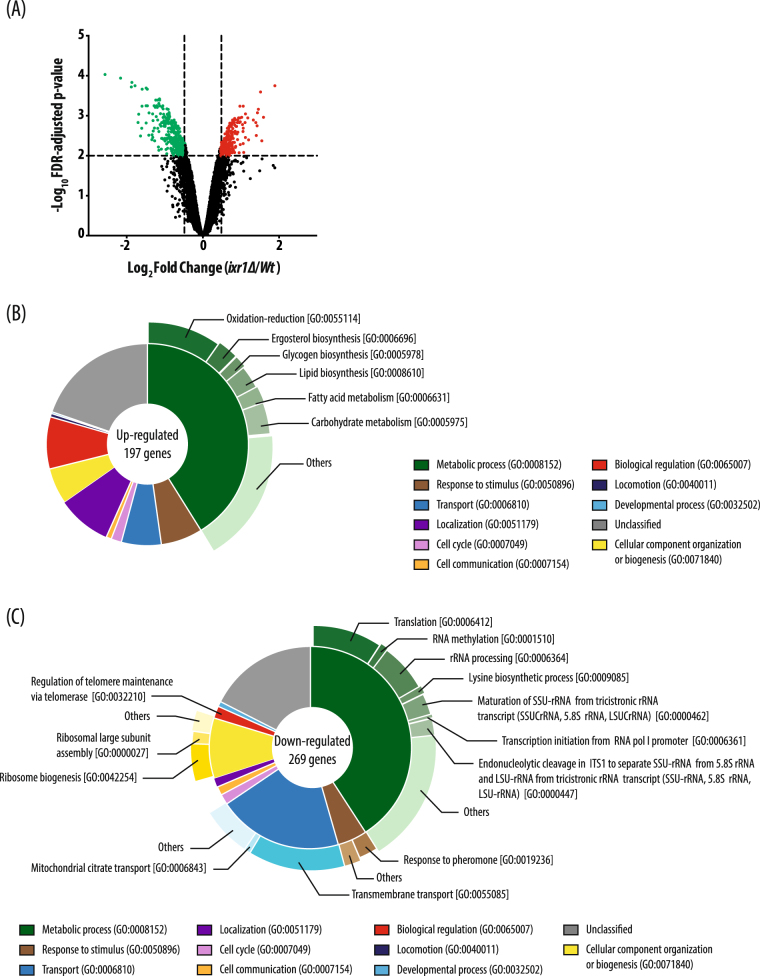
Transcriptome changes associated to *IXR1* deletion. (**A**) Volcano plot of transcriptome changes after *IXR1* deletion, depicting individual probe pK value (−log_10_) *versus* expression fold change (log_2_). Dotted lines represent on the logarithmic corresponding scale the p-value selected threshold (p < 0.05) and the fold change cut off threshold (>1.40). Upregulated genes are red and downregulated genes are green. (**B**) Distribution of functional categories over-represented (green sector) in the up-DEGs in *ixr1∆ versus* W303 strain. (**C**) Distribution of functional categories over-represented (green sector) in the down-DEGs in *ixr1∆ versus* W303 strain.

Variations in the transcriptome of the *ixr1Δ* strain compared to the isogenic W303 strain, carrying the wild type *IXR1* allele, were assessed as described in Materials and Methods. Of a total of *S*. *cerevisiae* 5744 probe sets, the GeneChip® Yeast-Genome-2.0 arrays showed that 499 were significantly changed (Fig. 1A); 197 genes being upregulated (Table S1) and 302 downregulated (Table S2). Functional distribution of upregulated genes analyzed with FUNSPEC shows that enriched groups include those involved in oxidation-reduction processes, response to stress, and lipid or carbohydrate metabolism (Fig. 1B). Most of the genes related to response to stress are genes expressed during anaerobic or hypoxic conditions. The data are in accordance with the function previously attributed to Ixr1 as a repressor of hypoxic genes under normoxic conditions^6–10^, or with its participation in the response to oxidative stress following H_2_O_2_ treatment^8^. Several differences between differentially expressed genes (DEGs), reported in our study in a W303 genetic background and a previous report on a BY4741 genetic background^10^, concern genes from the ergosterol biosynthetic pathway (*ERG28*, *NCP1 MCR1*, *ERG5*, *ERG24* and *ERG10*), which are downregulated in W303-*ixrΔ* but unaffected in BY474-*ixrΔ*^10^. These genes are regulated by 2 homologous transcriptional factors, Ecm22 and Upc2, that bind to their target promoters under normoxia or hypoxia, respectively^23^, and which also regulate Ixr1 expression^8^. Notoriously, this mechanism is functional in the W303 strain, but not in the BY4741 strain^23^, hence differences in Ixr1-dependent regulation of *ERG* genes in the two strains might be caused by their genetic background. Distribution of downregulated genes in enriched functional groups, analyzed with FUNSPEC, includes GO-terms associated with ribosome biogenesis, translation, metabolism of amino acids, membrane transport or ion channels (Fig. 1C).

**Figure 2:**
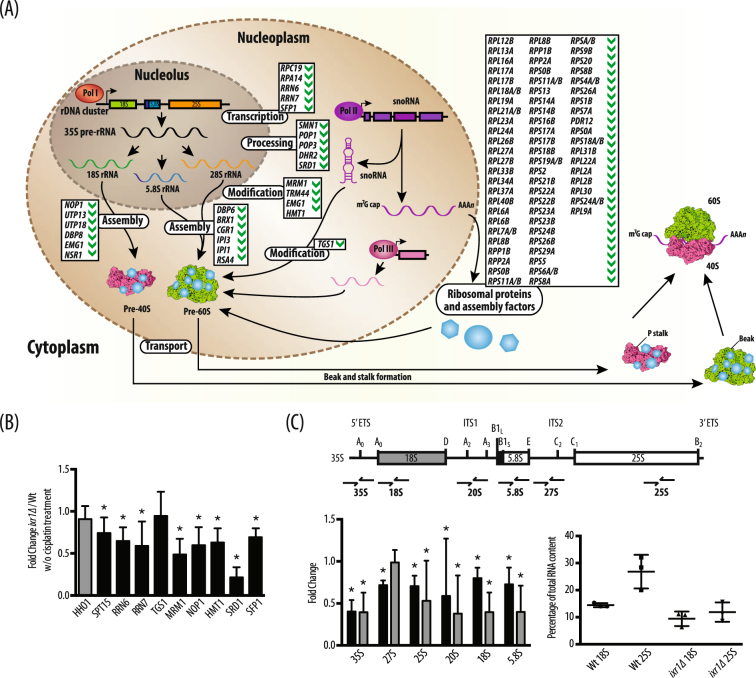
Ixr1 and ribosome biogenesis. (**A**) Genes downregulated after Ixr1 depletion associated to rRNA transcription, rRNA processing or ribosome assembly. (**B**) Validation of Ixr1 regulation of the main genes from this process by qPCR (**C**) Expression levels of rRNAs and their precursors. Upper scheme, relative positions of the primers designed for qPCR quantitation of the rRNAs and precursors. Lower left-part: fold changes in rRNAs and precursors expression in *ixr1∆ versus* W303.

After *IXR1* deletion, downregulation of genes related to rRNA, ribosomal proteins (RP), non-ribosomal proteins necessary for ribosome biogenesis (RiBi) and rRNA processing occurs (Table S2 and Fig. 2A). *SMN1*, *POP1* and *POP3* encode subunits of ribonuclease MRP, an enzyme that participates in pre-rRNA cleavage^24^; *POP1* and *POP3* encode components of the RNase P complex^24^. *DHR2* encodes a DEAH-box ATP-dependent RNA helicase and, like its homologue *DHR1*, it is required for 18S rRNA synthesis^25^. *SRD1* encodes a zinc finger protein involved in processing pre-rRNA to mature rRNA^26^. *NOP1*, *UTP13*, *UTP18*, *DBP8*, *EMG1*, *NSR1* encode assembly factors that function in the maturation of 40S ribosomal subunits^27^. *DBP6*, *BRX1*, *CGR1*, *IPI3*, *IPI1*, *RSA4* are involved in the maturation of 60S ribosomal subunits^27^. Other DEGs are *RPA14*, encoding subunit A14 of RNA Pol I^28^, *RPC19*, encoding RNA polymerase subunit AC19, a Pol I and Pol III subunit^29^, and *RPO26* encoding RNA polymerase subunit ABC23, common to RNA Pol I, Pol II and Pol III^30^. Genes encoding general transcription factors, which recruit RNA Pol I to the ≈150 tandem repeats of the rDNA transcription unit found on chromosome XII in *S*. *cerevisiae*, such as *RRN6* and *RRN7*, are also included^31^. *RRN6* and *RRN7* encode components of the core factor (CF), analogous to SL1 in mammals, necessary for the formation of the preinitiation complex (PIC) in the promoters of genes transcribed by RNA Pol I^31^. An important subset of genes regulated by Ixr1 is formed by RNA-methylases that are also related to ribosome biogenesis, e.g. *TGS1*, *MRM1*, *TRM44*, *NOP1* and *MGE1*, or RNP-methylases, e.g. *HMT1*^32–36^.

Data from transcriptome analysis were validated by qPCR. Several genes involved in ribosome biogenesis (*SPT15*, *RRN6*, *RRN7*, *TGS1*, *MRM1*, *NOP1*, *HMT1*, *SRD1* and *SFP1*) were selected and their mRNA levels determined in W303 and *ixr1∆* strains. Except for *TGS1*, the qPCR data confirmed the CHIP analysis (Fig. 2B).

Genome-wide analysis of Ixr1 binding to *S*. *cerevisiae* promoter regions by ChIP (Table S5) confirmed the transcriptome data. It is well known that experimentally discovered binding sites for TFs represent usually only a small percentage (5–30%) of their actual regulated targets, as reported in yeast^37^, plants^38–41^, and animals^42,43^. Ixr1 shows physical interactions with the upstream regions of genes, previously defined as positively or negatively regulated by Ixr1 in normoxia or hypoxia^8–10^. Among Ixr1 binding targets, taking into consideration upstream regions up to −1000 bp from the ATG, genes related to anaerobic growth, such as *YHR210C*, *PAU7*, *HEM13*, *YJR120W*, *DAN4*, *AUS1*; oxidative stress: *STB5*, *GTT1*, *ECM4*, *CTT1*, *HMX1*, *TSA1*; metabolism of sulfur containing compounds compounds: *CYS3*, *SUL2*; DNA repair: *RAD51*, *RAD54*, *EXO1*, *RNR4*; responsiveness to DNA damage or replicative stress: *CRP1*, *CAP2*, *NNR2*, *MRS4*, *IGD1*, *EDC1*, *YGR126W*, *TMA10*, *BCH1*, *YOR062C*, *PRE10*, *YBL029C-A*; rRNA and ribosome biogenesis: *TOD6*, *RPL23 A*, *MSI1*, *ENP1*, *RRP7*, *RSA4*, *THO1*, *RPL14B*, *NMD3*, *HCA4*, *RPL22A*, *UBP10*, *ECM23*, were found (descriptions of these genes and their concrete functions in these processes are included in Table S5). These results confirm and extend the role of Ixr1 as a transcriptional regulator in *S*. *cerevisiae*^6–10,12^.

**Figure 3:**
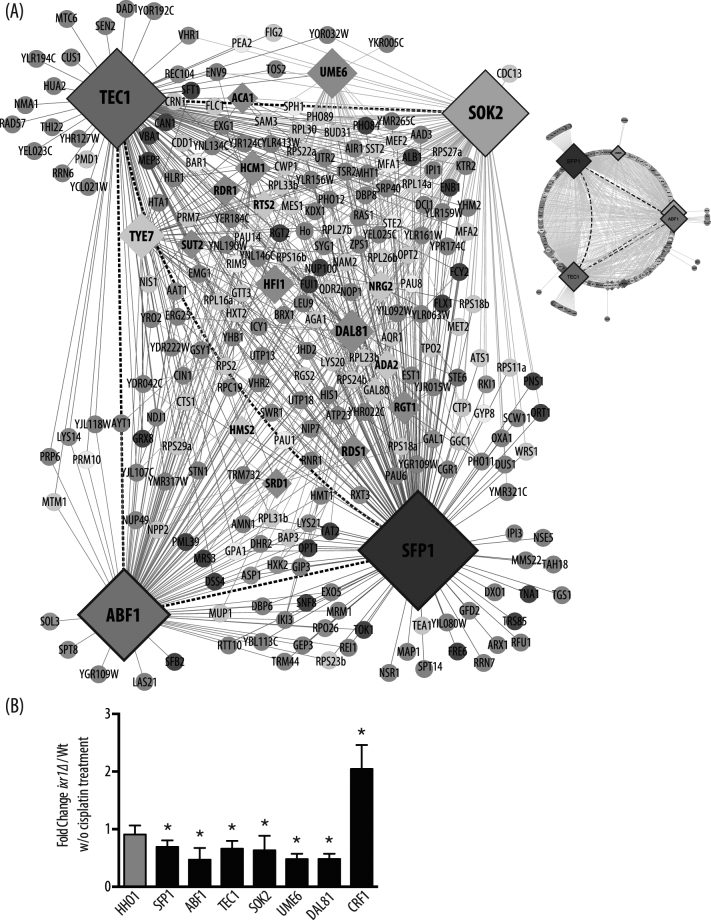
*IXR1* deletion affects transcription of other yeast general regulators. (**A**) Regulatory network showing the role that Ixr1 has, represented in circular or extended layouts of the major transcriptional regulators Tec1 (red), Sok2 (orange), Abf1 (green) and Sfp1 (dark blue). Dark dashed lines indicate direct nodes between general regulators and rhombus correspond to genes that encode transcriptional regulators. Genes related to translation [GO:0006412] are light green, ncRNA metabolism [GO:0034660] pink, transcription [GO:0006351] light blue, transport [GO:0006810] violet, hexose metabolism [GO:0019318] brown, sexual reproduction [GO:0019953] yellow, and others grey. (**B**) qPCR confirmation of the regulation exerted by Ixr1 on major nodes; analysis of *HHO1* expression was included as a non-regulated control. Wt, W303 strain; W/O, without cisplatin treatment.

Analysis of the DNA-array data (Table S2) also shows that Ixr1 positively regulates the transcription of a set of 33 yeast transcriptional regulators, which are downregulated in the *ixr1Δ* strain. We have constructed the regulation network that interconnects these 33 genes and their known targets, those also included in the Ixr1-dependent transcriptome shown in Fig. 3A. The major nodes are represented by yeast general regulators that notably are involved in the regulation of cell growth and cell cycle progression in response to nutrient availability, external stimuli or DNA damage. Sfp1 is a positive regulator of ribosomal protein (RP) gene transcription by RNA polymerase II, which closely links ribosome biogenesis to cell size^44^. It has been proposed that Sfp1 integrates information from nutrient- and stress-responsive signaling pathways to control RP gene expression^45^, and also mediates the DNA-damage response^46^. Abf1 is an essential general regulator that mediates many chromatin-related events, including nucleotide excision repair^47^ and transcriptional activation of the L2 ribosomal protein genes^48^. Tec1 links TORC1 and MAPK signaling pathways to coordinate cellular control in response to nutrients^49^. Sok2 is involved in the PKA signal transduction pathway^50,51^. Ume6 couples metabolic responses to nutritional cues related to the initiation and progression of meiosis^52–54^. Finally, Dal81 is a general positive regulator of genes involved in the utilization of poor nitrogen sources, such as γ-aminobutyric acid (GABA), leucine or allantoin^55^. Physical binding of Ixr1 to the promoters of these genes was not detected by ChIP analysis (Table S5), therefore regulation could be explained by indirect mechanisms caused by regulation of other intermediary DNA-binding effectors, or by other post transcriptional mechanisms controlling their mRNA levels. Alternatively, these genes could be considered as “transient targets” of Ixr1, defined as those regulated, but without detectable binding^41^.

qPCR measurements were made to validate the transcriptional regulation exerted by Ixr1 on the expression of these transcription factors representing the major nodes in the regulation network shown in Fig. 3A. These data, which included the analysis of genes *SFP1*, *ABF1*, *TEC1*, *SOK2*, *UME6*, and *DAL81*, confirmed the findings (Fig. 3B). Also included in this analysis was the *CRF1* gene that encodes a co-repressor protein of ribosomal protein (RP) genes and is upregulated in the *ixr1Δ* strain (Table S1) and therefore under the negative control exerted by Ixr1. Upregulation in the *ixr1Δ* strain was also confirmed by qPCR (Fig. 3B).

### Ixr1 control on transcription of Sfp1, rRNA, and genes involved in ribosome biogenesis, functionally affects rRNA levels

Synthesis of ribosomal components and their assembly is closely associated with cell growth and proliferation^27^; 90% of total cellular transcription is used during ribosome biogenesis of rapidly growing cells^56^. In *S*. *cerevisiae*, transcription is the major level of regulation of ribosome biogenesis^57,58^, involving all 3 nuclear RNA polymerases. As described above, *SFP1* is downregulated in the W303-*ixr1Δ* strain (Table S3). Sfp1 is a zinc finger protein that regulates transcription of ribosomal proteins and other genes related to ribosome biogenesis^45^, as also responses to DNA-damage, nutrient availability, cell cycle progression^46^ and cisplatin resistance^59^. It binds DNA directly at highly active RP genes and probably indirectly through Rap1 protein at others^60^.

To test whether the transcriptional regulation of genes related to rRNA transcription had a functional significance, we first measured 18S and 25S rRNAs levels by fluorescence assay (Fig. 2C), which showed a significant decrease in the *ixr1∆* strain in reference to W303. The levels of 18S, 5.8S, and 25S rRNAs were also measured, as well as those of their pre-processed forms 35S, 27S, 20S, by using qPCR and specific primers for each form (Table S7). The levels of these transcripts were always significantly higher in the wild type strain W303 than in the *ixr1∆* strain (Fig. 2C).

### Effect of *IXR1* deletion on transcriptional response to cisplatin treatment

The effects of cisplatin treatment upon the *S*. *cerevisiae* transcriptome have been previously reported^61^. The major functional group over-represented among downregulated genes is related to ribosome biogenesis, including genes involved in the maturation of SSU-rRNA from tricistronic rRNA transcript, or those participating in ribosomal small subunit assembly and/or rRNA export from the nucleus^61^. We have directly measured the cisplatin effect on the levels of 18S, 5.8S, and 25S rRNAs, and their pre-processed forms (35S, 27S, 20S) in the wild type strain W303, and results obtained (Figure S2) also confirmed the decrease observed in the previous transcriptome assay^61^.

**Figure 4:**
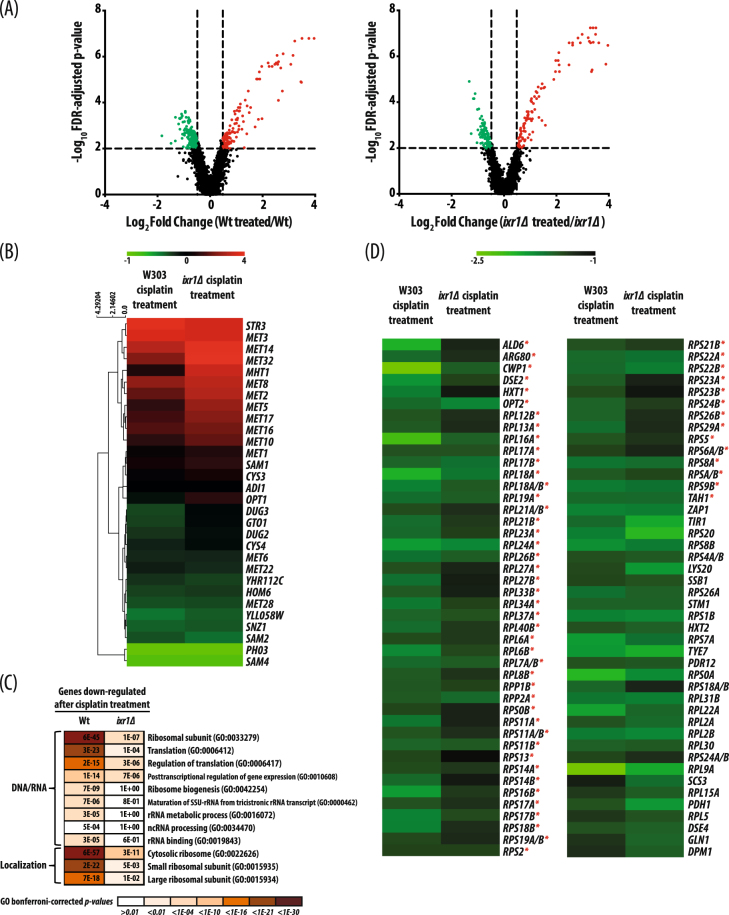
Effect of *IXR1* deletion in the response to cisplatin. (**A**) Volcano plots of cisplatin effect in W303 and *ixr1∆* strains, depicting individual probe p-value (−log_10_) *versus* expression fold change (log_2_). Dotted lines represent on the logarithmic corresponding scale the p-value selection threshold (p < 0.05) and the fold change cut-off threshold (>1.40). Upregulated genes are red and downregulated genes green. (**B**) Heat-map representing relative changes of expression (log_2_) of genes related to sulfur metabolism [GO:0006790] after cisplatin treatment in W303 and *ixr1∆* strains. (**C**) Comparison of the statistical significance of GO-term enrichment among downregulated genes in W303 and *ixr1∆* strains after cisplatin treatment. (**D**) Heat map representing relative changes (Log_2_) of expression of genes related to ribosome biogenesis after cisplatin treatment in W303 and *ixr1∆* strains. Red stars indicate statistically significan differences between the 2 strains (p > 0.01).

To more fully understand the role of Ixr1 in the response to cisplatin, we used transcriptome analysis to see how the deletion of *IXR1* affects this response. Significant changes as a consequence of cisplatin treatment occurred in both W303 and *ixr1∆* strains (Fig. 4A). The transcriptional activation of genes related to metabolism of sulfur compounds, including cysteine and methionine, is stronger in the *ixr1Δ* strain than in the W303 strain (Fig. 4B). However, expression of most genes related to ribosome biogenesis remain unchanged or with low change in the *ixr1∆* strain treated with cisplatin (Fig. 4C and D), indicating that Ixr1 is necessary in the ribosomal-gene response to this compound.

**Figure 5:**
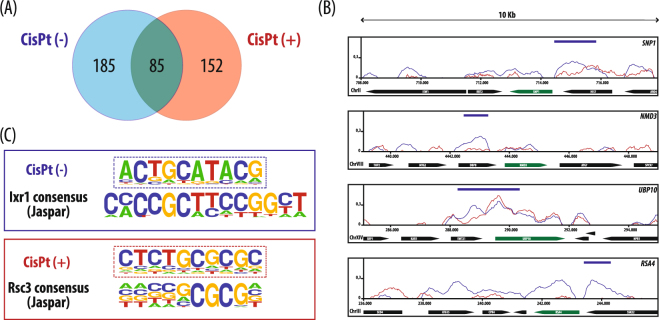
Changes in Ixr1 binding produced by cisplatin treatment. (**A**) Venn diagram of statistically significant Ixr1 binding peaks in promoter regions before (blue) and after (red) cisplatin treatment, obtained by ChIP-on-chip experiments. (**B**) Examples of Ixr1-peaks before (blue) and after (red) cisplatin treatment, obtained by ChIP-on-chip for the genes *SNP1*, *NMD3*, *UBP10* and *RSA4*. Y-axis represents signal values after input background subtraction. Bars at the top mark the peaks of interest reported by TAS. (**C**) Logo representation of *de novo* consensus sequences derived with HOMER from Ixr1 binding peaks related to promoter regions before and after cisplatin treatment, aligned with known consensus sequences in Jaspar database. (p-value: 1e−12, for motif significance according to HOMER).

DNA binding of Ixr1 in the presence of 600 μM cisplatin was analyzed with the same criteria used for the analysis of the data where the compound had not been used. Delimiting upstream regions up to −1000 bp from the ATG, 237 peaks were obtained. Only 85 Ixr1-binding peaks were on the same promoters found in the absence of cisplatin (Fig. 5A). A pool of 152 promoter peaks was exclusively detected in the presence of cisplatin treatment. Among these genes, the GO term “substrate-specific transmembrane transporter activity” [GO:0022891] is significantly enriched (p < 0.05) and includes 20 matches. This group includes hexose and amino acid transporters and other genes that have been related to detoxification processes. The latter group includes *FUI1*, involved in the transport of the cytotoxic nucleoside analog 5-fluorouridine^62^ or *PUT4*, related to the transport of the toxic proline analog, azetidine-2-carboxylate^63^; *FTR1* and *ZRT1*, related to the transport of metals^64,65^; *TPO1*, involved in exporting spermine and spermidine from the cell during oxidative stress^66^; finally and remarkably, *QDR2* encoding a protein that exports copper and also a range of other compounds, and which is required for resistance to cisplatin and other drugs^67^.

As previously explained, the effect of cisplatin on the transcription of genes related to ribosome biogenesis diminishes in the absence of Ixr1 (Fig. 4C and D). Peaks that are not formed in presence of cisplatin include important regulators for rRNA or ribosome biogenesis, such as *TOD6*, *ENP1*, *RRP7*, *NMD3*, *HCA4*, *UBP10*, *ECM23 or RSA4*^27^. The consensus for Ixr1 binding is found among the binding peaks seen in absence of cisplatin, whereas it is not found in the analysis of peaks exclusively formed after cisplatin treatment (Fig. 5C). Remarkably, up to 49% of the Ixr1 promoter binding peaks seen in presence of cisplatin contain a consensus sequence related to specific binding of the Rsc30 protein (Fig. 5C).

## Discussion

Our work demonstrates that Ixr1 controls ribosome biogenesis by direct binding to promoters of specific genes that regulate rRNA, RP and RiBi gene expression, but also by indirect regulation of specific transcriptional regulators, such as Sfp1 or Crf1; in these cases without observing direct binding of Ixr1 to their promoters. As a result of both direct and indirect mechanisms, *IXR1* deletion reduces 18S and 22S rRNA levels.

In eukaryotes, the target of rapamycin (TOR) signaling pathway promotes anabolic functions necessary for cell growth, while suppressing other catabolic processes, such as autophagy^68^. There are two effectors, the TOR complexes 1 and 2 (TORC1 and TORC2), which have functional specialization. TORC1 is sensitive to rapamycin, is related to nutrient signaling, and controls cell proliferation and ribosome biogenesis; TORC2, which is rapamycin insensitive, is associated with the control of actin cytoskeleton and cell cycle progression^69^. It is also involved in cell wall integrity and sphingolipid metabolism^70^. Considering that TOR activation promotes ribosomal biogenesis and Ixr1 controls transcription of genes related to this process, the intriguing question is how Ixr1 transcriptional control overlaps with the TOR signaling pathway. Indeed, other yeast HMG proteins are involved in TOR signaling, albeit by epigenetic mechanisms^71^.

Comparison of Ixr1 targets and TOR signaling pathway components shows overlap (Figure S1). It has also been suggested that TORC1 is activated by an abundance of leucine^72^; remarkably, Ixr1 is necessary for active transcription of genes encoding enzymes involved in the synthesis of leucine, isoleucine and valine^10^, which might contribute to the deactivation of TORC1 signaling in the absence of Ixr1. The TOR pathway controls DNA damage responses by regulating dNTP production^45,73,74^, which again connects to Ixr1 function, since Ixr1 regulates dNTP pools^22^. Although nitrogen activates the TOR signaling pathway^75^, optimal growth also requires a carbon source. In yeast, the cAMP/PKA pathway, which works on the basis of nutrients availability, growth, proliferation, metabolism, stress resistance, aging and morphogenesis, is activated by glucose^76^. Since TOR and cAMP/PKA are connected^77,78^, changes in glucose availability may also affect the final TOR targets. Deletion of *IXR1* (Fig. 4A) also affects the expression of genes encoding hexose transporters and Sok2, which is involved in the response to glucose and it is phosphorylated by PKA^50^.

**Figure 6:**
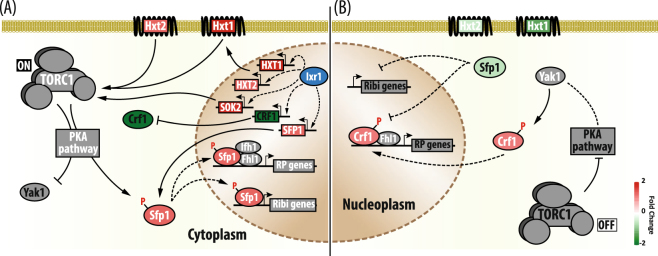
Speculative model about how connections of Ixr1 regulation, TOR and PKA signaling influence ribosome biogenesis. (**A**) Ribosome biogenesis activated by transcriptional regulation mediated by Ixr1 and post-translational modifications dependent on TOR and PKA signaling. (**B**) Decrease of ribosome biogenesis as anticipated from *IXR1* knockout or TOR inactivation. The color in the forms reflects Ixr1 transcriptional regulation, activation (red) or repression (green). The intensity of the color indicates the fold change *ixr1∆ versus* W303, according to the scale. White letters are used for activation of signal pathways and targets, and black letters for their inactivation.

From our data and in the context of published papers, a hypothetical model of cooperation between Ixr1-dependent and TOR-dependent mechanisms for maintaining ribosome biogenesis is proposed (Fig. 6). We found that when Ixr1 is functional, *SFP1* is actively transcribed (Fig. 6A). The subcellular localization of Sfp1, the master regulator of Ribi and RP genes, is regulated by both the cAMP/PKA and TOR network in response to nutritional and stress inputs^45^. In growing cells not limited by nutrient availability, the TOR1 pathway is also active and therefore Sfp1, the key regulator of RiBi and RP genes, localizes in the nucleus where it activates the transcription of these regulons^44,45,79^, thus allowing ribosome biogenesis, growth and proliferation (Fig. 6A). The other point of transcriptional activation mediated by Ixr1 upon the RP genes is through repression of the co-repressor Crf1 (Fig. 6A). Fhl1, a forkhead-like protein has a dual role as activator (in association with the Ifh1 co-activator) or repressor (in association with the Crf1 co-repressor) in the transcription of the RP genes^80–82^. Since Fhl1 is constitutively bound to the RP gene promoters, its activity depends on the presence of Ifh1 or Crf1. In growing cells, TOR keeps Crf1 inactive in the cytoplasm by repressing Yak1 kinase, possibly via a PKA-dependent route^81^, allowing expression of RP genes (Fig. 6A). In the absence of Ixr1, expression of the *SFP1* gene is reduced, as already noted (Fig. 6B). In response to carbon and nitrogen starvation, oxidative stress and inactivation of TOR signaling, Sfp1 rapidly translocates to the cytoplasm (Fig. 6B), and loses its function^44,45,79^. After TOR inactivation, phosphorylated Crf1 in the nucleus displaces Ifh1 (Fig. 6B), thereby inhibiting transcription of RP genes^81^. Moreover, the nuclear localization of both Fhl1 and Ifh1 is influenced by Sfp1^80^. Therefore, Ixr1 might also affect indirectly the subcellular localization of Ifh1 and Crf1 by affecting Sfp1 expression in addition to its effect on *CRF1* mRNA levels reported in our analysis (Fig. 6A).

After cisplatin treatment, the transcriptional activation of genes related to metabolism of sulfur compounds, including cysteine and methionine, is stronger in the *ixr1Δ* strain than in the W303 strain (Fig. 4B). Stimulation of a pathway of amino acid biosynthesis seems paradoxical in a situation in which the biosynthesis of ribosomal proteins is downregulated. However, specific sulfur-containing amino acids may be necessary to cope with oxidative stress, and their upregulation could favor cisplatin resistance in the null mutant by increasing chelating groups to immobilize the Pt compound, perhaps promote glutathione biosynthesis to favor anti-oxidant reactions, or favor cisplatin extrusion from the cell, through the formation of cisplatin-glutathione complexes. In support of this view, several mechanisms affecting cisplatin toxicity have been related to glutathione levels in human cells^83^.

We have shown that cisplatin treatment prevents Ixr1 binding to several promoters controlling ribosome biogenesis. In humans, the cytotoxic effect of cisplatin is attributed to diverse mechanisms, among which is reduced ribosome biogenesis^84,85^. Since cisplatin treatment mimics *IXR1* deletion in the control of transcription of genes involved in ribosome biogenesis, one possible explanation is that the Ixr1 protein binds to cisplatin-DNA adducts at other locations, being displaced from these promoters. Alternatively the formation of cisplatin-DNA adducts might induce chromatin remodelling and affect specific Ixr1-DNA binding. We have observed that the 37% of the Ixr1 promoter binding peaks seen in presence of cisplatin contain a consensus sequence related to the specific binding region of the Rsc30 protein (Fig. 5C). Rsc30 forms part of the *S*. *cerevisiae* ATP-dependent remodeling complex RSC (*Remodels the Structure of Chromatin*) that has 17 subunits^86^. RSC modulates access to chromatin, and therefore controls DNA metabolism, including replication, transcription, recombination, and DNA repair. RSC performs a large number of different remodeling activities, including exchange or incorporation of core histones or histone variants, eviction of histones or nucleosomes and repositioning or sliding of nucleosomes^87^. Interestingly, RSC complexes are also involved in the transcription of genes related to ribosome biogenesis^88^; also, strains lacking the subunits Rsc1 or Rsc2 are hypersensitive to a variety of DNA damaging agents^89–92^. The RSC complex participates in the inactivation of the TORC1 pathway in response to nitrogen starvation^93^. Considering all data, the existence of a putative interplay between Ixr1 binding and RSC-remodeling is a new question to be considered in future studies.

In summary, *IXR1* deletion diminishes transcription of ribosomal RNAs and genes encoding ribosomal proteins, or which are necessary for ribosome assembly, by direct and indirect mechanisms. Cisplatin treatment mimics the effect of *IXR1* deletion on rRNA and ribosomal gene transcription, and prevents Ixr1 binding to specific promoters of genes involved in these processes.

## Methods

### Cell culture and treatments

*S*. *cerevisiae* strain W303 (MATa *ade*2-1 *can*1-100 *leu*2-3,112 *trp*1-100 *ura*3-52) and its derivative, W303-*ixr1Δ* (previously described by^94^, have been used in the transcriptome experiments. *S*. *cerevisiae* strain Z1580 (MATa *ade2-1 trp1-1 can1-100 leu2-3*,*112 his3-11*,*15 ura3* GAL + psi + *IXR1:myc9::TRP1*) was obtained from Young’s lab^95^.

Three biological replicas of cultures and treatments were made. Yeast cells were cultured overnight in 10 mL of complete medium (SD) containing per liter: 6.7 g of bacto-yeast nitrogen base without amino acids (Difco, Franklin Lakes, New Jersey, USA); 40 mg of the following additives (w/v): histidine, leucine, adenine, uracil, lysine and tyrosine; 10 mg arginine, methionine and threonine; 30 mg tryptophan; 60 mg phenylalanine and isoleucine; and 2% glucose. For transcriptome analyses on the following day, the media were inoculated at an initial OD_600_ of 0.4 in 70 mL SD. Cells were grown in 250 mL Erlenmeyer flasks at 30 °C with agitation at 250 rpm. When cells had reached an OD_600_ of 0.6, the cultures from each strain were continued in 2 × 25 mL aliquots, i.e. control and cisplatin treated. A stock solution of cisplatin at 6 mM in dimethyl sulfoxide (DMSO) was prepared and the drug added to the cultures at a final concentration of 600 μM, an equivalent volume of DMSO being added to the control cultures. The cells were kept at 30 °C with agitation at 250 rpm for 4 h in darkness to prevent the inactivation of cisplatin. The concentration of cisplatin and the time course of the treatment had previously been established in pilot experiments with the selected yeast strains.

For ChIP-on-chip experiments, the cells were inoculated at an initial OD_600_ of 0.1 in 200 mL SD and grown in 1 L Erlenmeyer flasks at 30 °C with 250 rpm agitation. When cells had reached an OD_600_ of 0.6, cisplatin was added to the cultures at a final concentration of 600 μM. The time-course of the treatment was applied as explained for transcriptome analyses.

### RNA preparation and transcriptomic microarray analysis

RNA was extracted from a number of cells corresponding to an OD_600_ of 3 with the AurumTM Total RNA Mini Kit (Bio-Rad). Concentration and purity of RNA was measured using the ratio R = A_260_/A_280_ (always in the range of 1.7 < R < 2.1). RNA integrity was also measured by the RIN parameter (RNA Integrity Number) with a 2100 Bioanalyzer (Agilent Technologies, Inc. Santa Clara, CA 95051-7201USA). RIN was close to 9 in all the samples, which is considered high-quality extraction^96^.

Twelve GeneChip® Yeast-Genome-2.0 arrays from Affymetrix Inc. (Wycombe. United Kingdom) were used and processed in the GeneChip® System with Autoloader from Affymetrix Inc. (Wycombe, UK). We started from 10 ng total RNA from each sample for successive cDNA, aRNA generation, labeling with biotin and fragmentation using the GeneChip® 3′ IVT Express Kit. RNA fragmentation was monitored with a 2100 Bioanalyzer (Agilent Technologies, Inc. Santa Clara, CA 95051-7201, USA), by selecting conditions producing fragments from 35 to 200 nt, with a majority between 100–120 nt. Hybridization, washes and staining were done with the GeneChip® HT Hybridization, Wash and Stain Kit. (Ambion, Inc. Affymetrix). The kit includes RNA Poly-A controls (lys, phe, thr and dap) from *Bacillus subtilis* to monitor the target labeling process, which serve as sensitivity indicators of target preparation and labeling efficiency. They also include the hybridization controls, comprised of a mixture of biotinylated and fragmented RNA of bioB, bioC, bioD (genes from the biosynthesis of biotin in *Escherichia coli*) and Cre (recombinase from bacteriophage P1). These controls monitor hybridization, and the washing and staining steps. Control Oligo B2 was included to provide alignment signals for image analysis.

Image capture and preliminary data analysis were carried out with Affymetrix® Expression Console™ software (v1.1). After RMA normalization of raw data from 3 biological repeats using the Affymetrix algorithm, the normalized data were analyzed using the web-suite Babelomics (v4.3)^97^. Statistical analyses to identify differentially expressed genes (DEGs) used the LIMMA (linear models for microarray data) test^98^. The FDR (False Discovery Rate) was used to correct values for multiple comparisons^99^. Statistical significant DEGs were considered those with FDR < 0.01 and a fold change of ≥1.4 in the comparisons. The original and normalized data from this study are uploaded in Gene Expression Omnibus database (http://www.ncbi.nlm.nih.gov/geo/info/ linking.html), the accession number of the series being GSE84569. The processed data and DEG identifications are shown in Tables S1–S4.

### Chromatin immunoprecipitation and ChIP-on-chip analyses

Chromatin immunoprecipitation experiments were carried out as previously described^95^, with minor modifications. Briefly, 200 mL Z1580 yeast culture were grown until an OD_600_ of ≈0.9–1 had been reached. Cross-linking involved adding 1% formaldehyde to the cultures and incubation at room temperature for 20 min, at which time 125 mM glycine was added and cultures incubated for a further 5 min. Cells were harvested and washed 4 times with 50 mL Tris-HCl saline buffer (20 mM Tris-HCl, pH 7,5, 150 mM NaCl) at 4 °C. Cell breakage was done in 800 µL lysis buffer (50 mM HEPES-KOH, pH 7,5, 140 mM NaCl, 1 mM EDTA, 1% Triton X-100, 0,1% sodium deoxycholate, 2x complete protease inhibitor cocktail from Roche, and 2x complete phosphatase inhibitor cocktail from Roche) with glass beads, and cell extracts were sonicated for 5 min in 10 sec on/59 sec off cycles (the chromatin was sheared into average sized 400 bp fragments). Dynabeads (Invitrogen) were used for immunoprecipitation, with anti-(c-Myc) antibodies (sc47694; Santa Cruz Biotechnology) and being used for specific Ixr1-c-Myc immunoprecipitation. Negative controls were done with rabbit IgG immunoprecipitation. Samples were washed 3 times in 1 mL lysis buffer, 3 times in 1 mL high salt lysis buffer (50 mM HEPES-KOH, pH 7.5, 500 mM NaCl, 1 mM EDTA, 1% Triton X-100, 0.1% sodium deoxycholate), 3 times in 1 mL wash buffer (10 mM Tris-HCl, pH 8, 250 mM LiCl, 0.5% NP-40, 0.5% sodium deoxycholate, 1 mM EDTA), and once with 1 mL TE Buffer (10 mM Tris-HCl, pH 8, 1 mM EDTA). Immunoprecipitants were recovered in 250 µL elution buffer (50 mM Tris-HCl, pH 8, 10 mM EDTA, 1% SDS) and treated overnight with 30 µL proteinase K (20 mg/mL, NewEngland Biolabs). Immunoprecipitated DNAs were cleaned the next day with USB PrepEase DNA Clean-Up kit (USB). The next steps rigorously followed the manufacturer’s (Affymetrix) instructions at: (http://cmgm.stanford.edu/pan/section_html/GE/protocols/Chromatin%20Immunoprecipitation%20Assay%20Protocol.pdf). Specific enrichment after inmmunoprecipitation was checked by qPCR (data not shown) to identify promoter regions of *TIR1*, *IXR1*, *ROX1* and *HEM13*, already known to bind Ixr1^7–9^. In all cases, >8-fold enrichments were obtained for IP samples compared to the IgG controls.

GeneChip® *S*. *cerevisiae* Tiling 1.0 R arrays from Affymetrix Inc. (Wycombe,UK) were used with 3 biological repeats and processed in the GeneChip® System with Autoloader also from Affymetrix. Control Oligo B2 was included to provide alignment signals for image analysis. Image caption and preliminary data analysis used Affymetrix® Expression Console™ software (v1.1). ChIP-on-chip raw data from Affymetrix GCOS software were analyzed using Affymetrix Tiling Analysis Software (TAS) v1.1.03 (http://www.affymetrix.com/support/developer/downloads/TilingArrayTools/index.affx).

A 2-sample analysis was conducted for both untreated and cisplatin treated cultures using specific immunoprecipitated DNA samples (from Ixr1-c-Myc tagged samples) as the ‘experimental’ group, and 2 genome-DNA fragmented and amplified samples as the ‘control’ group. Data were normalized using built-in quartile normalization and probe-level analysis with perfect match (PM) probes. Ixr1 protein occupancy profiles were visualized with Affymetrix Integrated Genome Browser (IGB). Interval analyses used TAS software with a minimum run of 150 bp a maximum gap of 250 bp, and a p-value cutoff of 10^−4^. Bed file conversions used UCSC (University of California Santa Cruz) tools (https://genome.ucsc.edu). *Bed file analyses used ChIpSeek tools (http://chipseek.cgu.edu.tw), as described by Chen *et al*. (2014).

Our original and normalized data have been uploaded into the Gene Expression Omnibus database (http://www.ncbi.nlm.nih.gov/geo/info/linking.html); the accession number of the series is GSE101080, and processed data are available in Tables S5–S6.

### Data mining

Gene descriptions and comparative analyses of lists from DEGs were obtained from Yeast Mine (http://yeastmine.yeastgenome.org/yeastmine).

Functional distribution of genes in the differentially regulated clusters was analyzed using FunSpec (http://funspec.ccbr.utoronto.ca/), developed by Robinson^100^ and PANTHER (http://pantherdb.org), as previously reported^101^. The MIPS Functional Catalogue Database (FunCatDB) was used in the analyses (http://mips.helmholtz-muenchen.de/proj/funcatDB/), for which p < 0.01 was selected. Hierarchical and k-means clustering were estimated with the Multiple Array Viewer package (MeV, v10.2), using the ‘organize genes’ option and default options of ‘Euclidean distance’ and 100 runs. Motif was analyzed with HOMER (Hypergeometric Optimization of Motif EnRichment)^102^ in the ChIPSeek suite (http://chipseek.cgu.edu.tw/index.py)^103^, and YEASTRACT (Yeast Search for Transcriptional Regulators And Consensus Tracking) (http://www.yeastract.com)^104^. Regulatory pathways were constructed by YEASTRACT and Cytoscape (http://www.cytoscape.org)^105^.

### Analysis of the expression by qPCR

Methods and procedures have been previously described^61^. The ECO Real-Time PCR System was used (Illumina) and calculations were made by the 2^−∆∆Ct^ method^106^. Three independent RNA extractions were assayed for each strain or condition. The list of primers is given in Table S7. RNA levels of the selected genes were corrected by the geometric mean of the mRNA level of *TAF10*, a gene previously verified to be constitutive in the assayed conditions and not affected by *IXR1* deletion. *HHO1* was used as the negative control, and was also unaffected in the *ixr1∆ strain*. A t-test was used to find statistically significant differences between control and cisplatin treated samples (p < 0.05).

### Comparison of rRNA levels in W303 and *ixr1Δ* strains

Total RNA was extracted as previously described^61^ from yeast cultures collected at logarithmic growth phase (OD_600_ of ≈ 0.9–1). Two biological and 2 technical repeats of each were measured for the W303 and *ixr1Δ* strains. Prior to RNA analysis, RNA-containing pellets were resuspended in RNase-free water (Sigma-Aldrich) and incubated at 75 °C for 5 min to resolve secondary structures.

The relative amounts of 18S and 25S rRNAs per unit of total RNA were estimated by analyzing total RNA with an Agilent 2100 Bioanalyze with its RNA 6000 Nano kit (Agilent Technologies, Palo Alto, USA). Total RNA and RIN ratios were quantified under the Agilent eukaryotic total RNA program as previously described^96^. 18S and 25S rRNAs quantitation was calculated from the area under the peaks in reference to total RNA in the sample. Relative amounts of 5, 8S 18S, 25S rRNAs, as well as their precursor forms (35S, 27S and 20S), were measured by qRT-PCR following the procedures described in section 2.5. Specific primers were designed for each type (Table S7), and their relative positions indicated in Fig. 2C.

## Electronic supplementary material

Supplementary Information

Dataset 1

Dataset 2

Dataset 3

Dataset 4

Dataset 5

Dataset 6
