# Supplementary material for: Ixr1 Regulates Ribosomal Gene Transcription and Yeast Response to Cisplatin
Source: Sci Rep. 2018 Feb 15;8:3090. doi: 10.1038/s41598-018-21439-1 (PMC5814428; doi:10.1038/s41598-018-21439-1)
Supplement: Supplementary file 1 — Supplementary Information [file 41598_2018_21439_MOESM1_ESM.pdf]

SUPPLEMENTARY INFORMATION TO

Ixr1 Regulates Ribosomal Gene Transcription and Yeast Response to Cisplatin

Ángel Vizoso-Vázquez, Mónica Lamas-Maceiras, M. Isabel González-Siso and M. Esperanza Cerdán*

Universidade da Coruña, Grupo EXPRELA, Centro de Investigacións Científicas Avanzadas (CICA), Facultade de Ciencias, 15071 A Coruña, Spain.

Runing Head: Ixr1 master rRNA regulator and cisplatin response

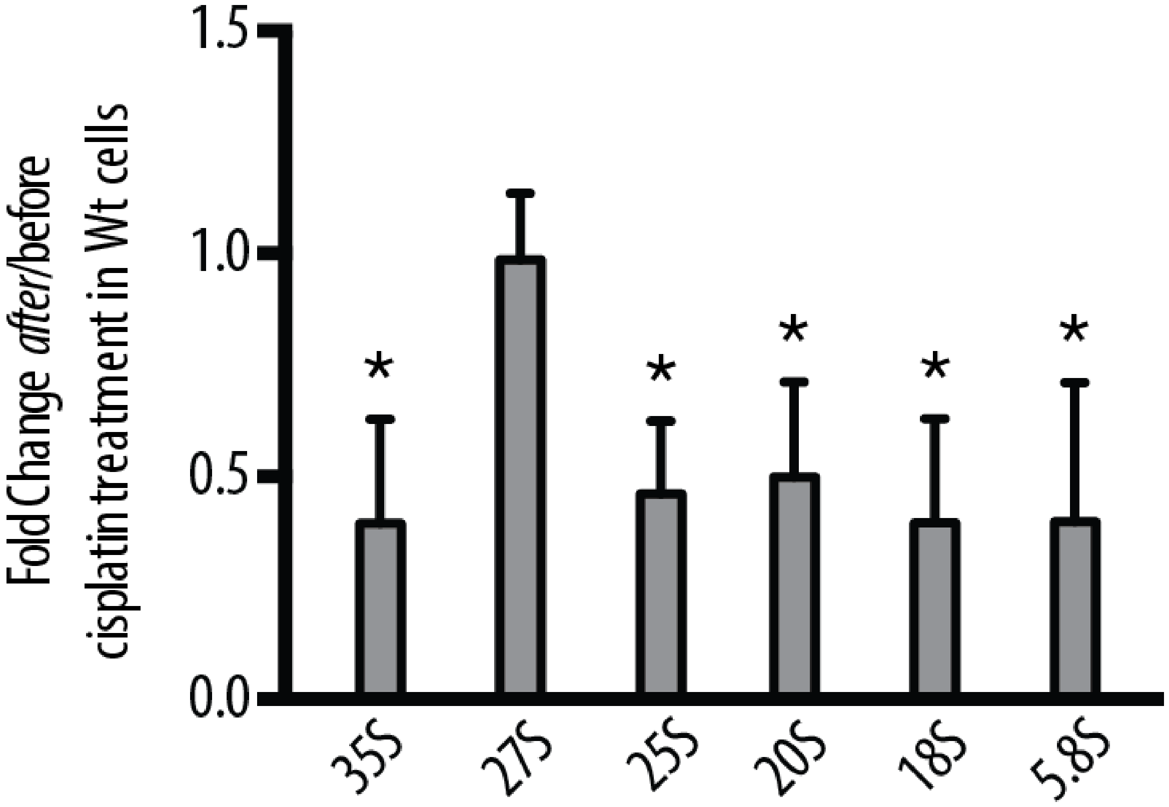

Figure S1. Fold changes in rRNAs and precursors expression change in W303 cells after cisplatin treatment *versus* untreated cells. Relative positions of the primers designed for qPCR quantitation of the rRNAs and precursors have been depicted in Figure 3C.

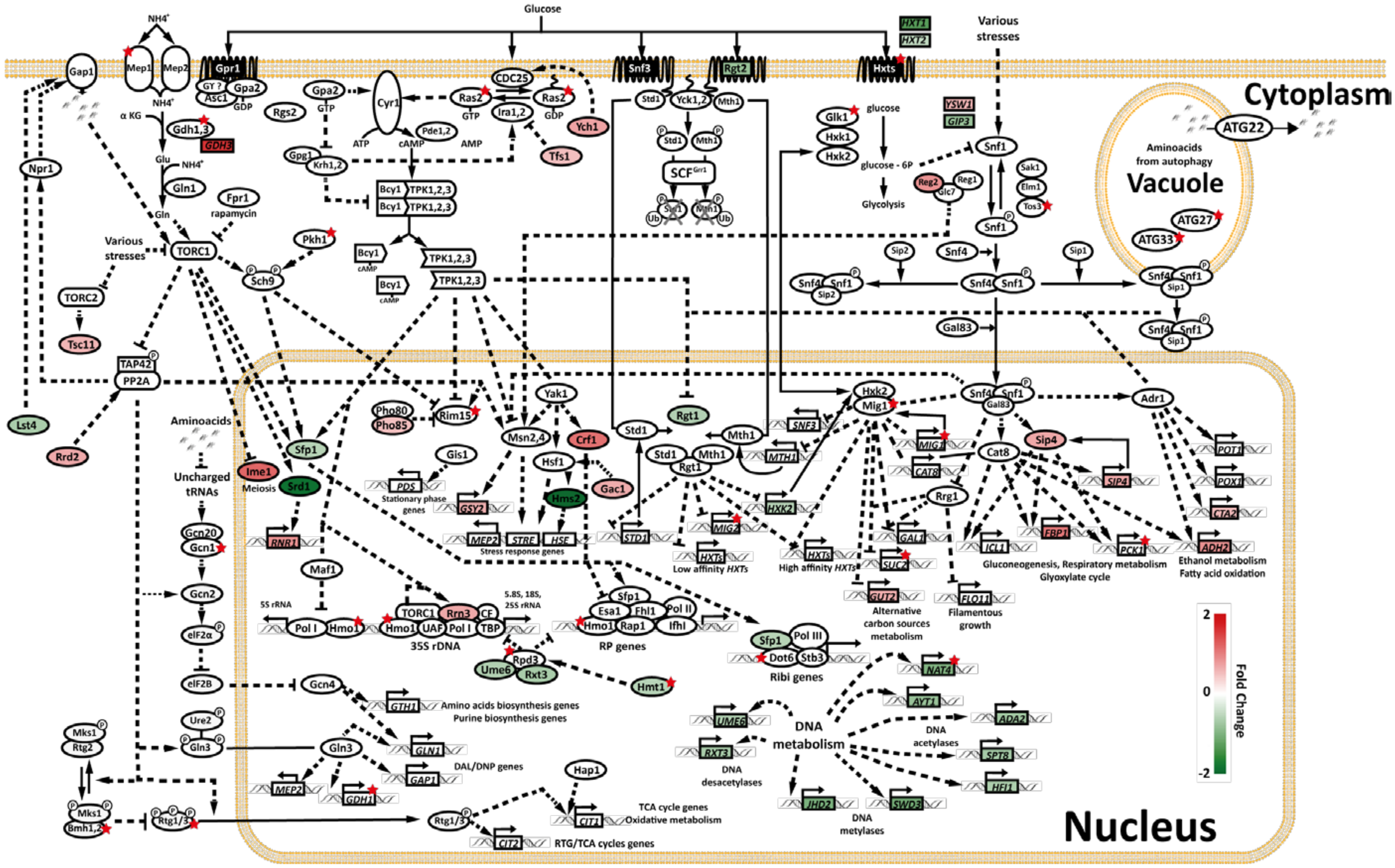

Figure S2. Schematic view (modified from [78, 107] showing how *IXR1* deletion alters the transcription of several genes encoding proteins involved in TOR and other nutrient sensing pathways. In the signal pathways, genes are represented by squares and proteins by ellipses. Color shading of either squares or ellipses represents that these components of the pathways are transcriptionally influenced by Ixr1. The color intensity code represents the fold change values (*ixr1*Δ *versus* W303), as indicated on the scale. Red stars are genes in whose promoter regions an Ixr1 binding site was detected by ChIP on chip experiments.

Table S7.-Oligonucleotides used in this study

Oligo Name	Sequence	Gene	Strand	Position
AVV220	AGAACTTGGCGATTGCTGACA	*ROX1*	W	-408
AVV221	AAGACCGTTACATTACGCAAAGTG	*ROX1*	C	-275
AVV222	CATACACATCGTGCTTAGCGATC	*IXR1*	W	-526
AVV223	CCCATTCGTTCTCTCACCAAG	*IXR1*	C	-376
AVV224	CATAAAGGGTCTCTTTCACCTATACG	*TIR1*	W	-273
AVV225	CTTCACTTTTTTCTCTGTCAAGGG	*TIR1*	C	-178
AVV226	TCAAACCATTTCCTGCGGAG	*HEM13*	W	-539
AVV227	TGCCTATGACGGTAATCCCAG	*HEM13*	C	-406
Primer A ChIP	GTTTCCCAGTCACGGTCNNNNNNNNN	-	-	-
Primer B ChIP	GTTTCCCAGTCACGGTC	-	-	-
AVV35q	GACCACAAGTAAGGGCAAGAA	*HHO1*	W	+28
AVV36q	GCCTTGGAAGTTGATTTCTCC	*HHO1*	C	+89
AVV37q	GAGGAGATTCTAGAGATGATGGACA	*TAF10*	W	+223
AVV38q	AGTCTATTACTGCATCGGGAATG	*TAF10*	C	+283
AVV379	CACTAGTTCATCAGTTCGTATGACAA	*SFP1*	W	+732
AVV380	GGCCATGTTATTCTGCAGGT	*SFP1*	C	+801
AVV381	TCATGGTAATGACAGCGGTAAC	*ABF1*	W	+630
AVV382	TTTCGTCATTTGGGTATGGAC	*ABF1*	C	+696
AVV383	CTGGACTGGGTGCTAAATCG	*TEC1*	W	+224
AVV384	TCTGCTTGTCAGTGAACGTAGC	*TEC1*	C	+292
AVV385	TTCCTGAACAGTGGCCGTA	*SOK2*	W	+188
AVV386	GCAGTTGCTGTTGAGACTGG	*SOK2*	C	+253
AVV387	TGCTCCAGAACAACAACAGC	*UME6*	W	+1936
AVV388	GCGTTTCCAACTGACCTTCT	*UME6*	C	+2003
AVV389	GCTTATTTTGCCACGGAAAT	*DAL81*	W	+1861
AVV390	CAGTTCCTTTGGAGTTTGAGGA	*DAL81*	C	+1935
AVV391	AACTGTCGTCCTTTCATCCAA	*CRF1*	W	+111
AVV392	ATAGAGGGGTCCCAAAGAGC	*CRF1*	C	+170
AVV363	CCAGCAACTACTTTCCAGAGTG	*SPT15*	W	+106
AVV364	GCGGAGGTGTCTTTTTCAGA	*SPT15*	C	+177
AVV365	CCCCACCGGTATAGAGACAA	*RRN6*	W	+1513
AVV366	CCTCCTCTCTCCGAGATTCA	*RRN6*	C	+1577
AVV367	CCCAGACAAACCGACTTCTAGT	*RRN7*	W	+1071
AVV368	CCATCCACTTCAAAAACTCTAGG	*RRN7*	C	+1144
AVV369	AGTGTGACGCCCGAAAGA	*TGS1*	W	+229
AVV370	CAGTATTCTTTCGGCATTTGG	*TGS1*	C	+306
AVV371	CTGGGGGTTGACTTCATTGT	*MRM1*	W	+793
AVV372	GCTTGTCTTGGACACCACAG	*MRM1*	C	+865
AVV373	CCCCAGGCAAGAAAGTTTTA	*NOP1*	W	+494
AVV374	CCAACAACATCTGAAACGTGA	*NOP1*	C	+566
AVV375	TGGGTTACTTTCTACTATACGAGTCCA	*HMT1*	W	+401
AVV376	CCGCCTTCTACCAAATAGTGG	*HMT1*	C	+476
AVV377	CATGCAATTCCGAGAGACG	*SRD1*	W	+323
AVV378	TGCACAACTATGGTAGCCTTCA	*SRD1*	C	+390
AVV406	CCGGGGCCTAGTTTAGAGAGAAG	*RDN37-1 (37S)*	W	+6682

Table S7.-Oligonucleotides used in this study (continued)

AVV407	AATACATGTTTTTACCCGGATCATAG	*RDN37-1 (37S)*	C	+6773
AVV408	GCTGGCCTTTTCATTGGATG	*RDN37-1 (27S)*	W	+3077
AVV409	CCGTACTTGCATTATACCTCAAGC	*RDN37-1 (27S)*	C	+3150
AVV410	TGGTCAGAAAGTGATGTTGACGC	*RDN37-1 (25S)*	W	+5410
AVV411	CTTAAGAGAGTCATAGTTACTCCCGC	*RDN37-1 (25S)*	C	+5523
AVV412	CGGTGAGAGATTTCTGTGCTTTTG	*RDN37-1 (20S)*	W	+2657
AVV413	TGAAAACTCCACAGTGTGTTGTATTG	*RDN37-1 (20S)*	C	+2729
AVV414	TACAGTGAAACTGCGAATGGCTC	*RDN37-1 (18S)*	W	+777
AVV415	GCTCTAGAATTACCACAGTTATACCATG	*RDN37-1 (18S)*	C	+866
AVV416	GCATCGATGAAGAACGCAGC	*RDN37-1 (5.8S)*	W	+2892
AVV417	AATGTGCGTTCAAAGATTCGATG	*RDN37-1 (5.8S)*	C	+2975

Sea also datasets

Table S1. DEGs upregulated in *ixr1Δ* compared with W303 in absence of cisplatin treatment.

Table S2. DEGs downregulated in *ixr1Δ* compared with W303 in absence of cisplatin treatment.

Table S3. DEGs upregulated in *ixr1Δ* compared with W303 after cisplatin treatment.

Table S4. DEGs downregulated in *ixr1Δ* compared with W303 after cisplatin treatment.

Table S5. Ixr1 DNA-binding in absence of cisplatin.

Table S6. Ixr1 DNA-binding after cisplatin treatment.
